# Measuring Artificial Sweeteners Toxicity Using a Bioluminescent Bacterial Panel

**DOI:** 10.3390/molecules23102454

**Published:** 2018-09-25

**Authors:** Dorin Harpaz, Loo Pin Yeo, Francesca Cecchini, Trish H. P. Koon, Ariel Kushmaro, Alfred I. Y. Tok, Robert S. Marks, Evgeni Eltzov

**Affiliations:** 1 School of Material Science and Engineering, Nanyang Technology University, 50 Nanyang Avenue, Singapore 639798, Singapore; DORIN001@e.ntu.edu.sg (D.H.); yeolp@ntu.edu.sg (L.P.Y.); MIYTok@ntu.edu.sg (A.I.Y.T.); 2 Avram and Stella Goldstein-Goren, Department of Biotechnology Engineering, Faculty of Engineering Sciences, Ben Gurion University of the Negev, Beer-Sheva 84105, Israel; arielkus@bgu.ac.il; 3 Institute for Sports Research (ISR), Nanyang Technology University and Loughborough University, Nanyang Avenue, Singapore 639798, Singapore; 4 TURVAL Laboratories, Ltd. (Laboratori Turval Italia Srl), via J. Linussio 51, 33100 Udine, Italy; cecchini@turval.com; 5 Department of Obstetrics and Gynaecology, KK Women’s and Children’s Hospital, 100 Bukit Timah Road, Singapore 229899, Singapore; trishkoon@gmail.com; 6 School of Science and Technology, Singapore University of Social Sciences, 463 Clementi Road, Singapore 599494, Singapore; 7 The National Institute for Biotechnology in the Negev, Ben-Gurion University of the Negev, Beer-Sheva 84105, Israel; 8 The Ilse Katz Centre for Meso and Nanoscale Science and Technology, Ben-Gurion University of the Negev, Beer-Sheva 84105, Israel; 9 Agriculture Research Organization (ARO), Volcani Centre, Rishon LeTsiyon 15159, Israel

**Keywords:** artificial sweeteners, sport supplements, bioluminescent bacteria, toxic effect, gut microbiota, environmental pollutants

## Abstract

Artificial sweeteners have become increasingly controversial due to their questionable influence on consumers’ health. They are introduced in most foods and many consume this added ingredient without their knowledge. Currently, there is still no consensus regarding the health consequences of artificial sweeteners intake as they have not been fully investigated. Consumption of artificial sweeteners has been linked with adverse effects such as cancer, weight gain, metabolic disorders, type-2 diabetes and alteration of gut microbiota activity. Moreover, artificial sweeteners have been identified as emerging environmental pollutants, and can be found in receiving waters, i.e., surface waters, groundwater aquifers and drinking waters. In this study, the relative toxicity of six FDA-approved artificial sweeteners (aspartame, sucralose, saccharine, neotame, advantame and acesulfame potassium-k (ace-k)) and that of ten sport supplements containing these artificial sweeteners, were tested using genetically modified bioluminescent bacteria from *E. coli*. The bioluminescent bacteria, which luminesce when they detect toxicants, act as a sensing model representative of the complex microbial system. Both induced luminescent signals and bacterial growth were measured. Toxic effects were found when the bacteria were exposed to certain concentrations of the artificial sweeteners. In the bioluminescence activity assay, two toxicity response patterns were observed, namely, the induction and inhibition of the bioluminescent signal. An inhibition response pattern may be observed in the response of sucralose in all the tested strains: TV1061 (MLIC = 1 mg/mL), DPD2544 (MLIC = 50 mg/mL) and DPD2794 (MLIC = 100 mg/mL). It is also observed in neotame in the DPD2544 (MLIC = 2 mg/mL) strain. On the other hand, the induction response pattern may be observed in its response in saccharin in TV1061 (MLIndC = 5 mg/mL) and DPD2794 (MLIndC = 5 mg/mL) strains, aspartame in DPD2794 (MLIndC = 4 mg/mL) strain, and ace-k in DPD2794 (MLIndC = 10 mg/mL) strain. The results of this study may help in understanding the relative toxicity of artificial sweeteners on *E. coli*, a sensing model representative of the gut bacteria. Furthermore, the tested bioluminescent bacterial panel can potentially be used for detecting artificial sweeteners in the environment, using a specific mode-of-action pattern.

## 1. Introduction

Artificial sweeteners are an important class of sugar substitutes known as high-intensity sweeteners (HIS), also referred to as non-nutritive sweeteners (NSS) or as non-caloric sweeteners (NCS) [1]. The Food and Drug Authority (FDA) has approved the use of six artificial sweeteners, which includes aspartame, sucralose, saccharin, advantame, neotame and acesulfame potassium-k (ace-k), in food and beverages [2]. The recent EU legislation has also approved of these artificial sweeteners [3]. Artificial sweeteners provide a sweeter taste than sugar and also enhance food flavor, while contributing very little to energy intake [4]. These sweeteners are most commonly used as food additives [5]. Many different population groups consume the added ingredient, with or without their knowledge. This is especially common with athletes who devote full-time care to their diet, which often include sport supplements to improve their physical performance in trainings and competitions [6]. In several registered products’ patents [7,8,9], it is clearly stated that artificial sweeteners are added to electrolyte drinks and food supplements [10,11,12,13,14]. As a result, the average consumption of artificial sweeteners is higher in athletes and any potential health risks involved would also be more significant. 

The health risks of artificial sweeteners consumption is still a highly controversial topic [15]. Artificial sweeteners have allegedly been linked to adverse effects such as cancer, weight gain, metabolic disorders, migraines, type-2 diabetes, vascular events, preterm delivery, kidney function disorders, liver antioxidant system, hepatotoxicity, immune system disruptions and alteration of gut microbiota activity [16,17]. Although these potential health problems have long been studied, a firm conclusion has yet to be reached on these allegations due to a lack of consistent evidence. Subsequent human studies failed to show a direct connection to cancer risk [18,19]. Other studies, however, have shown association with kidney function decline [20] and vascular risk factors [21]. Consumption of artificial sweeteners as food additives has been promoted as a prevention strategy against obesity as well as a diet for weight loss as they replace the high-calorie sweeteners. Studies have compared a diet of artificial sweeteners versus no artificial sweeteners and artificial sweeteners versus traditional sugars, with results showing greater weight loss and better weight management in an artificial sweetener diet [22,23]. However, the converse has also been proven true [24,25]. It was shown that consuming diet soda results in more weight gain than consuming naturally-sweetened soda [26]. In another study, rats given artificial sweeteners showed steadily increasing caloric intake, increased body weight, and increased adiposity [27]. Since the 1980s, there have been studies reporting associations between artificial sweeteners and alteration in bacterial composition. Subsequent studies, investigating the possible effects of artificial sweeteners on the gut microbiota system, presented controversial results. A recent study has shown that the intake of artificial sweeteners such as lactitol or maltitol increased some beneficial bacteria such as lactobacillus in the gut system [28]. A second study concluded that artificial sweeteners induce glucose intolerance. Mice were fed with artificial sweeteners in drinking water and demonstrated gut microbiota changes [29]. Another related study was conducted on pigs fed with artificial sweeteners but it was concluded that there is a selective effect on the gut microbiota [30]. Moreover, artificial sweeteners have been identified as emerging environmental pollutants [31,32]. They are resistant to wastewater treatment processes, therefore they are continuously introduced into the water environments [33]. Several environmental studies have confirmed the widespread distribution of ace-k, saccharin and sucralose in the water cycle [34,35,36,37,38,39]. Concentrations of ace-k and sucralose up to the μg L^−1^ range can be found in receiving waters, i.e., surface waters, groundwater aquifers and drinking waters. Such concentrations are among the highest known for anthropogenic trace pollutants [31,40].

Typically, the toxicological evidence is derived from studies in appropriate animal models, and possibly from human trials. The compounds can be evaluated across a wide range of exposures, including duration and persistence of exposures [41]. Other associated manifestations of toxicity in humans may be identified from the case reports and epidemiological studies after product marketing. However, all of these approaches are time-consuming and expensive. Thus, there is a demand for fast and simple approaches that will provide toxicity evaluation of the artificial sweeteners. Progress in the genetic engineering field allows not only the “tailoring” of microorganisms for determining the identity of the target analyte but also allows the monitoring of the biological activity of these chemicals by analyzing different cell responses (e.g., gene expression, metabolic activity, viability). In this study, bacteria engineered to luminesce after exposure to certain stresses were used [42]. The bioluminescent bacteria, which luminesce when they detect toxicants, act as a sensing model representative of the complex microbial system. The relative toxicity of the six FDA approved pure artificial sweeteners (aspartame, sucralose, saccharine, neotame, advantame and ace-k) and 10 sport supplements containing artificial sweeteners were tested using three different *E. coli* strains (TV1061, DPD2544 and DPD2794) of genetically modified bioluminescent bacteria sensitive to the various stresses (e.g., cytotoxicity, amino acids availability, genotoxicity, accordingly). Luminescence was easily measured using a sensitive photodetector unaffected by variable background signals [43]. The bioreporter bacteria developed in this study used luciferase as the reporter gene, which provided a sensitive and simple detection method for gene expression and regulation [44]. An additional advantage of a bacterial luciferase-based bioassay is the ability to express a whole luciferase operon that produces a luminescent cell without any additions or external existing sources, thus allowing for real-time monitoring of gene expression [42]. Expressed effectively in different strains, the bacterial *lux* system has been used for sensing various compounds such as heavy metals [45,46,47], androgen-like [48,49], active oxygen [50], endocrine disrupting chemicals [51], phenolics [52] and other environmental pollutants [53,54,55,56,57]. The simplicity and biological relevance of bacterial bioreporter assays makes them attractive as a rapid and cheap monitoring method of the presence of toxicants in water, air, soil and food samples [58].

## 2. Results

### 2.1. Artificial Sweeteners Toxicity and Viability Effect

**Figure 1:**
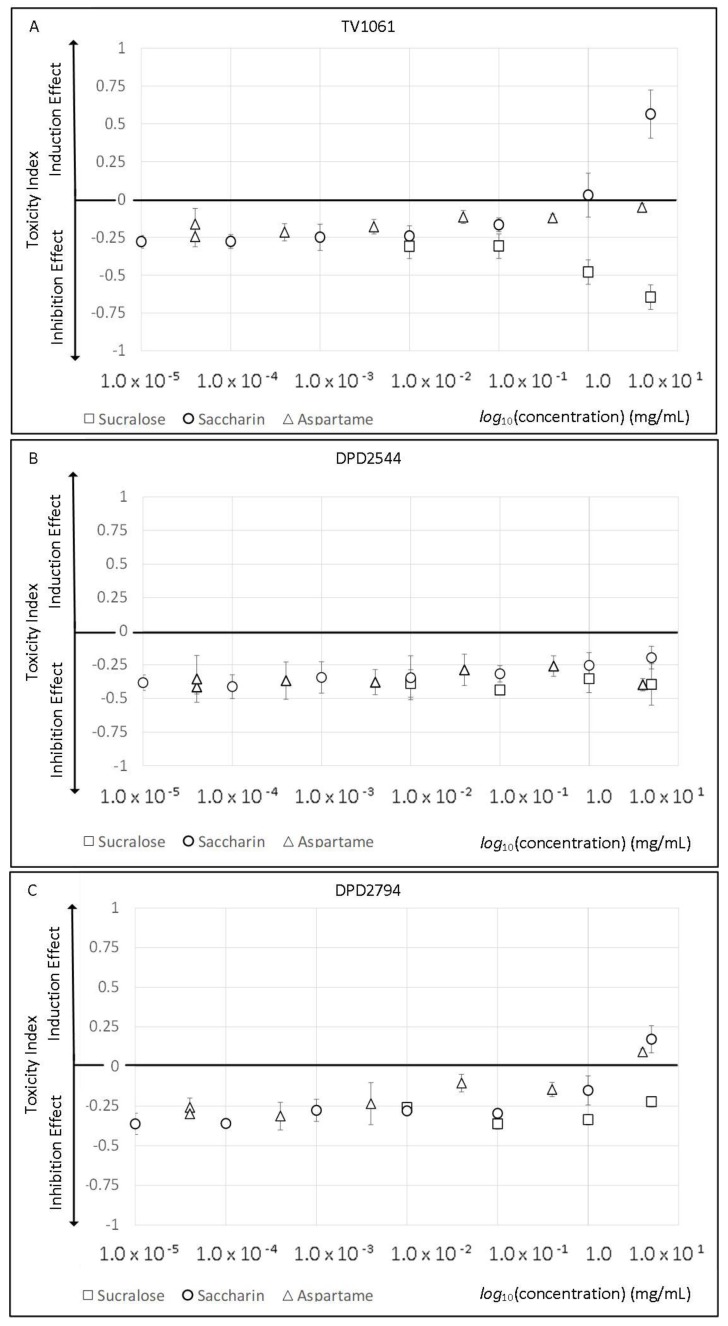
Artificial sweeteners toxicity. The toxicity index of different artificial sweeteners on the three tested bioluminescent bacteria strains: (**A**) TV1061; (**B**) DPD2544; (**C**) DPD2794. A strong induction response pattern may be observed in the response of the TV1061 strain to saccharin and DPD2794 strain to aspartame and saccharin. In addition, a strong inhibition response pattern may be observed in the response of the TV1061 strain to sucralose.

The biological effect of the artificial sweeteners on the bioluminescent bacteria was quantified using a toxicity index. This index describes the response ratios between treated and untreated microorganisms and may provide information about the possible toxicity of the artificial sweeteners. Figure 1 shows three different response patterns of the bioreporter bacteria to the tested chemicals. The first toxicity response pattern is signal induction, where increased chemical concentration induced bacterial luminescence. This may be observed in the response of the TV1061 strain to saccharin (Figure 1A) and DPD2794 strain to aspartame and saccharin (Figure 1C). In all of these cases, dose dependent responses were observed, where greater chemical concentrations produced higher inducing effect. The second toxicity response pattern is inhibition, where increased chemical concentrations decreased cells luminescence (response of the TV1061 strain to sucralose (Figure 1A)). As in the previous case, in an inhibition pattern, higher concentrations have a stronger biological effect. For the third response pattern, no visible effect was observed through all tested concentrations (Figure 1B). 

During this study, we exposed different bioluminescent bacterial strains to various artificial sweeteners (Table 1), and compared their minimum luminescent inhibition concentration (MLIC), minimum luminescence induction concentration (MLIndC), minimum growth inhibition concentration (MGIC) and minimum growth induction concentration (MGIndC). The logic behind this method is that, during luminescent activation, the promoters fuse to the *lux* reporter genes and will not only show the possible toxic effects of the artificial sweeteners but will also create a specific pattern. This allows us to infer the mode of action of the sport supplements through the induced bioluminescence pattern of specific bioreporter bacteria. From all the tested artificial sweeteners, only sucralose and neotame inhibited bioluminescent responses of the bioreporter bacteria (Table 1). Neotame reduces light response only in the DPD2544 strain while sucralose inhibits it in all the tested strains. Furthermore, in sucralose, this inhibition effect was observed not only with luminescence but also with bacterial growth (Figure S1 presented in Supplmentary Data). While the cells’ growth rates were affected by the same MGIC of 50 mg/mL in all strains, the luminescent signals were affected by different sweetener concentrations depending on the strains used. The light response of the DPD2794, SOS-dependent bioluminescence strain was inhibited by a sucralose concentration that was two-fold higher than that needed for the TV1061 cytotoxic strain. Interestingly, an induction effect was observed only for the cases of TV1061 with saccharin and neotame, and DPD2794 with aspartame, saccharin and ace-k.

In general, from all tested strains, TV1061 was the most susceptible to artificial sweeteners. The lowest inhibition and induction concentrations that resulted in a toxic response were observed with sucralose (1 mg/mL) and neotame (2 mg/mL), respectively. A light induction effect was observed only within TV1061 and DPD2794 strains. Furthermore, 5 mg/mL saccharin induced TV1061 luminescence while also showing a growth inhibition effect. In general, several induction and growth patterns may be observed, e.g., luminescence induction (DPD2794 with aspartame, saccharin, ace-K, and TV1061 with neotame), growth induction (TV1061 with advantame), and the combination of luminescence induction with growth inhibition (TV1061 with saccharin). From all the tested additives, only advantame induced growth without any visible effect on the luminescence signal. For the other tested chemicals, the MGIC parameter could not be estimated, with the maximum achievable concentration not showing any visible effect in all tested strains. To conclude, for all tested artificial sweeteners, the luminescence and growth effect pattern were either fully inhibition or induction, without any case of a combination of both toxicity response patterns within the same sample.

### 2.2. Sport Supplements Toxicity and Viability Effect

The response patterns of the bioluminescent bacteria strains to the chemicals present in sport supplements were also tested and the results are presented in Table 2. Different response patterns were obtained for each tested strain. The response pattern of the bacteria strains to the sports supplements were contrary to their response to the artificial sweeteners. The DPD2544 luminescence signal was inhibited by all of the sport supplements. Simultaneously, the luminescence signal of this strain also showed induction response albeit at lower concentrations of the sport supplement. In all samples, the MLIndC values (pg/mL) were 1000-fold lower than the MLIC values (ng/mL). Nevertheless, the sport supplements did not have any effect on the DPD2544 growth rate (Table 2). Conversely, the other two bacteria strains were inhibited only when exposed to a specific sport supplement, DPD2794 with SS3 and TV1061 with SS7.

The bacterial strains responded differently to the varying sport supplements, with four response patterns observed. In the first response pattern, only cell bioluminescence (MLIndC) was induced. In this case, MGIC or MGIndC parameters cannot be estimated as the maximum achievable concentration did not have any visible effect. MLIndC, however, was quantitative. Such response pattern was observed when the TV1061 strain was exposed to SS1, SS5, SS7 and SS9 sport supplements. In the second response pattern, in addition to the bioluminescence induction or inhibition, the bacteria growth rates were also affected (Figure S2 presented in Supplmentary Data). This is observed in the response of the DPD2794 to SS3 (MLIndC with MGIC) or SS5 (MLIC with MGIndC) (Table 2). In the third response pattern, only the growth rate of the bioreporter bacteria was affected by the sport supplements. While the supplements either inhibited (SS1, SS2, SS5, SS7) or induced (SS3, SS4, SS6, SS8, SS10) the growth rates of TV1061 and DPD2794 strains, only SS9 did not show any visible effect on the bacteria growth rate. In the last pattern, more than two trends in the bacterial responses were observed. For example, when SS7 was exposed to TV1061, it not only induced bacterial growth rates but also increased and decreased (at different concentrations) the cells bioluminescence.

**Figure 2:**
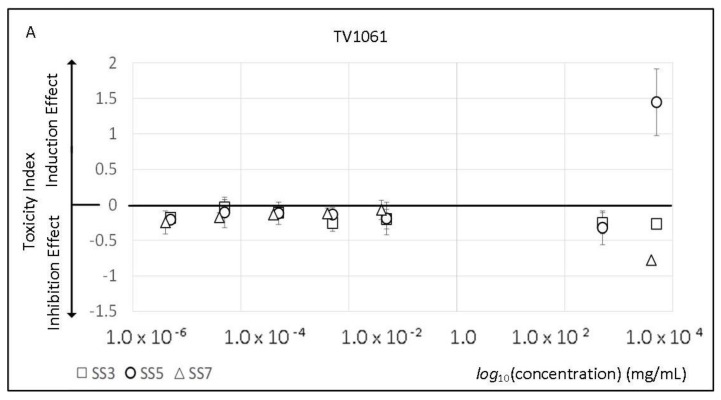

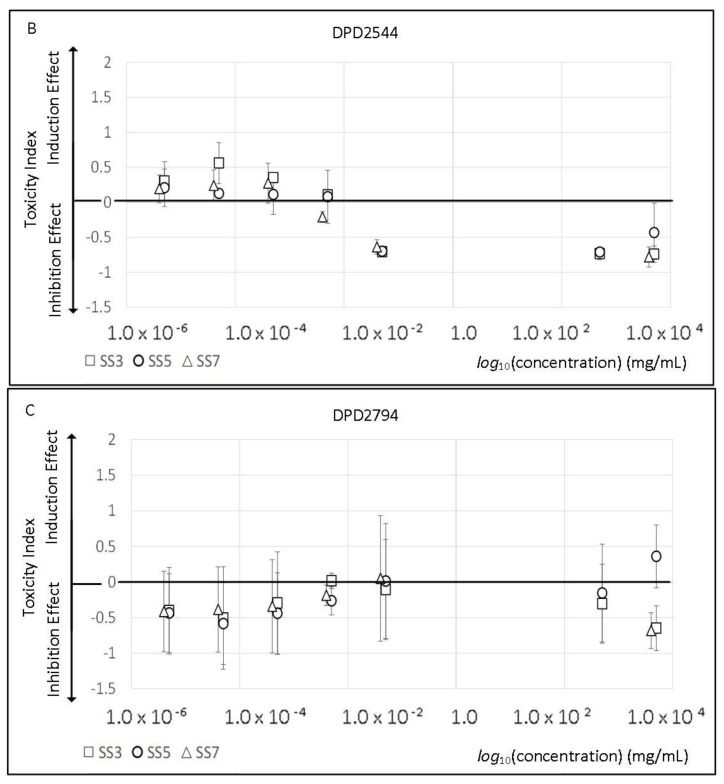
Sport supplements’ toxicity. Toxicity index of different sport supplements on the three tested bioluminescent bacteria strains: (**A**) TV1061; (**B**) DPD2544; (**C**) DPD2794.

The changes in the cell bioluminescence when exposed to the different concentrations of the three sport supplements (SS3, SS5, and SS7) are presented in Figure 2. The response of strain TV1061 (with heat-shock gene *grp*E fused to *lux* gene) to different sport additives (Figure 2A) resembles its response pattern to the artificial sweeteners (Figure 1A), whereby increasing concentrations of the sport supplements in the tested sample increased its biological effect on the TV061 strains in a dose-dependent manner. For example, at higher SS3 concentrations, the cell luminescence increased, whereas the SS7 showed increasing inhibition effect. The response of strain DPD2544 is also very similar to the cells’ reaction to the artificial sweeteners (Figure 2B). The main difference is that, at lower concentrations of sport supplements, the bacterial luminescence signals are induced, while, at higher concentrations, they are inhibitory. The DPD2794 also responded in a dose-dependent manner to the sport supplements, whereby an induction response in bioluminescence was observed in SS5 and an inhibition response was observed in SS3 and SS7. In general, at lower concentrations, the intensity of the signal received in response to the sport supplements was slightly lower than those produced by the exposure to the artificial sweeteners. However, at higher sample concentrations, the sport supplements showed a stronger induction or inhibition effect.

## 3. Discussion

### 3.1. Artificial Sweeteners’ Toxicity and Viability Effect

For decades, the food, beverages, and other industries have used artificial sweeteners as sugar substitutes for those who are diabetic and/or obese. Industries highlight the beneficial aspects of artificial sweeteners’ use, such as tooth friendliness, increased quality of life for diabetics and weight control [59]. However, in addition to the environmental pollution issues [31], there has been much evidence about the possible negative impact sugar substitutes contribute to human health [16,17,18,19,21,24,25,26,27]. However, the total consumption of artificial sweeteners in foods has only increased among people of all ages, with 28% of the total population consuming them [60]. For the consumers’ safety, it is necessary to control the content of sweeteners in foods. Several analytical methods (including high-performance liquid chromatography, ion chromatography, thin-layer chromatography, gas chromatography, capillary electrophoresis, flow-injection analysis, electroanalysis and spectroscopy) can determine sweeteners individually and in mixtures. However, there still remains the challenge of developing stable, reliable and robust methods for the determination of artificial sweeteners in complex food matrices and their putative toxic effect [59]. Moreover, our diet has a direct effect on the body’s microbiome, which not only plays important physiological roles but also reduces susceptibility to many pathophysiological conditions [61]. Thus, the microbiome may serve as a hub, channeling the effects of one’s diet onto the host’s health and propensity to disease. Artificial sweeteners, which are commonly found in dietary supplements, may be subjected to the same interactions with the microbiome and thus consequently exert their effects on the host [62]. To date, diverse methods (e.g., qPCR [63], turbidity [64], selective culturing [65], next-generation sequencing (NGS) [29]) across different species have been used for the determination of potential effects of the artificial sweeteners on microbiome. However, all such technologies are complicated, highly expensive and time-consuming. Given this situation, there is a need for a fast and simple application for the evaluation and characterization of the effects that artificial sweeteners (e.g., advantame, neotame, ace-K, aspartame, saccharin, and sucralose) have on the prokaryotic cells. For example, a previous study demonstrated the use of a whole cell microbial amperometric sensor, using an immobilized *Bacillus subtilis* cells for the detection of aspartame [66].

In this study, we demonstrate the use of a panel of indicator bacteria that is able to detect active compounds at subinhibitory concentrations and to predict the mode of action of these chemicals from their bioluminescence responses. We use the expression of the *lux* gene under the control of different stress promoters that are responsible for regulatory networks in the indicator bacteria. Three different strains were exposed to the commercial artificial sweeteners for the determination of their possible toxic effects (Table 1). The inhibition effect on the whole bioreporter panel was observed only with the exposure to sucralose. Previous studies have shown that bacteria do not utilize sucralose as a carbon source [67] and that the substitution of glucose with sucralose in agar medium produced a total inhibition of growth of several strains [68]. In this study, sucralose induced bacterial growth at the highest tested concentration. A possible explanation is that sucralose, which was added to the medium containing all of the nutrients required for cell growth, did not replace the already available carbon sources. Thus, it was not a limiting factor for the bacterial growth processes. On the other hand, however, sucralose repressed luminescence in all the bioreporter bacteria. The MLIC values were not only different for each strain tested (Table 1), but the strains’ kinetic responses also differed (Figure 1). In the tested concentration range, the highest inhibition effect was observed with TV1061 strain (1 mg/mL), and then with DPD2544 (50 mg/mL) and DPD2794 (100 mg/mL). Figure 1A demonstrates that only the TV1061 strain showed an increasing inhibition effect with higher tested concentrations. Such an inhibition pattern indicates that the sucralose mode of action is not affecting cyto/genotoxicity or fatty acid synthesis pathways. Indeed, sucralose was subjected to a full battery of in vitro and in vivo mutagenicity and clastogenicity studies, and no evidence indicated that sucralose have the genotoxic potential to induce genetic effects [69].

In addition to sucralose, the DPD2544 strain was also inhibited by neotame, an artificial sweetener with a very similar structure to aspartame but with higher sweetening power [70]. Toxicity of neotame was previously reported, at doses higher than its admissible daily intake [71]. In this study, the neotame concentrations tested were of lower concentrations but still induced TV1061 and inhibited DPD2544 luminescences. The possible reason for these results is the capability of the bioreporter bacteria to be affected by sub-active concentrations of the chemicals. It appears that some compounds (e.g., antibiotics [72]), when used at sub-toxic concentrations, may activate or repress gene transcription, which is distinct from their biological effects. Another possible reason is that the effective neotame concentration in this study was still two-fold higher than in real food samples [73]. Other toxicity tests did not evaluate the toxicity at such concentrations. The fact that neotame did not have any effect on bacterial growth reinforced these findings.

Acesulfame K is one of the most used artificial sweeteners in the world and is “generally regarded as safe” (GRAS) by the Food and Drug Administration of the United States of America [71]. However, reports on the genotoxicity testing of ace-K are contradictory. It was found to be genotoxic and clastogenic in mice [74]; non-mutagenic in a mammalian cell [74]; and non-cytotoxic and non-genotoxic both in “in vivo” and “in vitro” experiments [75]. In this study, ace-K induced luminescence only in genotoxic sensitive bacteria (DPD2794) indicating its possible genotoxicity.

As in the case of ace-K, aspartame also induced luminescence only with the genotoxicity sensitive strain. Aspartame is a low-calorie sweetener used to sweeten a variety of low and reduced calorie foods and beverages including low-calorie tabletop sweetener as well as in gums, breakfast cereals, and other dry products [76]. Aspartame has been extensively evaluated for genotoxic effects in microbial, cell culture and animal models [77,78]. These studies have shown evidence of induction of chromosomal damage in vitro [77]. Indeed, in our case, DPD2794 strain, not only showed luminescence induction effects but also showed dose-dependency (higher aspartame concentrations produced stronger cells response) (Figure 1C). Thus, these results enforce the previous data on aspartame genotoxicity to the *E. coli* strains.

In this study, amongst all of the tested artificial sweeteners, the strongest induction effect was observed with saccharin (Table 1). Saccharin is the oldest chemical sugar substitute and the best researched of all sweeteners [79], but it is still one of the most controversial food additives. Many studies have shown that saccharin may act as a weak mutagen [78] or produce cytotoxic effects [80]. Similar to these studies, our findings showed that saccharin induced the same luminescence responses in both cytotoxic and genotoxic bacteria (Table 1). Nevertheless, TV1061 not only showed much greater (three times more) responses than DPD2794, but also the growth rates of this strain was inhibited (Figure 1 and Figure 2). The results suggest higher cytotoxic than genotoxic effects of saccharin on the bacteria. In summary, two conclusions may be observed from these results. Firstly, different artificial sweeteners exhibit different toxicity types of effects and create specific response patterns. The second conclusion is that the bacterial responses were correlated to the results of previous toxicity studies and bacteria may be used as a toxicity evaluation tool.

### 3.2. Sport Supplements Toxicity and Viability Effect

Nutrition has always been perceived as an integral component affecting physical performances in sport competitions. The understanding of human metabolism and sport physiology shows a direct correlation between the performance in sports and the manipulation in nutrient intake. Thus, during the last decade, a large variety of sport supplements have been widespread and used routinely by athletes. However, the side effects of these sport supplements have yet to be fully elucidated due to the absence of compelling regulation and considerable variation in concentrations, terminology, and combinations of these products. Nevertheless, a wide range of commercial sports supplements are still available, with the majority of them containing artificial sweeteners. Numerous studies have evaluated artificial sweeteners toxicity and their effect on the human health. However, in general, they are concerned only with their addition into food products, with only a few examining their effect on sports supplements. In this study, ten different commercially available sports supplements were tested (Table 3). They were dissolved and exposed to the bioreporter bacterial panel, for toxicity evaluation. Each sport supplement mixture contains a variety of different compounds, but all of them include the addition of an artificial sweetener, either sucralose or/and ace-k, to sweeten the supplement flavour.

Similar to the artificial sweeteners’ toxicity results, the bioreporter panel responded differently to each tested sport supplement. Only when exposed to SS4, SS8, and SS10 mixtures did the bioreporter panel show a similar response pattern (Table 2). Due to the complexity of the commercial mixture, it is difficult to determine whether the responses were affected by the added artificial sweetener or whether it is due to the presence of another component. However, it is still important to examine their induction or inhibition effect on the bioreporter panel. For example, the bioluminescence of DPD2544 strain was induced and inhibited by all supplements, while the inhibitory concentrations were three-fold higher than the inducing concentrations. The DPD2544 strain exhibited a similar inhibition effect when it was exposed to sucralose, the same artificial sweetener used in most of the tested sport supplement mixtures (Table 1). However, the similarity in the response pattern to the sport supplement SS6 (a mixture not containing sucralose), which has no visible effects on the growth rates, indicates that the possible toxicity effect was produced by another component. Previously, DPD2544 was used as a bioreporter for the determination of “general toxicity” of several environmental contaminants, and its induction indicated interruptions in the fatty acid biosynthesis pathways [53]. The dose-dependent effect (induction at lower concentrations and inhibition at higher concentrations) of the sport supplements on the DPD2544 strain (Figure 2) suggests the same cytotoxicity mechanisms.

Such effect was also observed with the TV1061 strain, a bacterium sensitive to general cytotoxic damages. The sensitivity and reliability of this strain have been proven in many different applications for air [81], soil [82] and water [83] toxicity monitoring. In this study, the induction effect of SS1, SS5, SS7, and SS10 supplements on TV1061 indicates the activation of the cytotoxicity repair mechanisms in the cells, and therefore provides data on their possible toxicity mechanisms. The greatest effect on the bacterial panel was observed with the SS7 sport supplement mixture, where it not only induced and inhibited light responses in all strains, but also decreased growth rates. Similar to previous results, in all strains, the bioluminescence induction response pattern was observed at lower concentrations than the inhibition response pattern. For example, the presence of SS7 induced bioluminescence at three-fold or one-fold lower than the inhibitory concentrations in DPD2544 and TV1061, respectively (Table 2). DPD2544 has shown similar response patterns for all the tested mixtures, even in cases where no growth effects were observed (e.g., SS9). This strain is harboring the fusion *lux* genes with operon *fabA*, a gene responsible for the formation of a double bond in fatty acids used in the membrane of *E. coli.* Activation of this promoter is triggered by fatty acid starvation events caused by cell membrane damages [84]. Thus, DPD2544 may be used as a tool for monitoring internal cellular mechanisms that may be interrupted by consumption of sports supplements. The fact that the sport supplements triggered bacterial responses without any effect on the cell growth rates also helps to determine their toxicity grades (low in this case).

In contrast to DPD2544, cytotoxicity or genotoxicity effects (represented by growth and light changes) were observed at much higher concentrations for all tested supplements in other bacteria strains. For example, DPD2544 bacteria cells exposed to SS1, SS5, and SS8 were induced at nine-fold lower concentrations than with that of the TV1061 strain. Interestingly, the same strains were induced and inhibited simultaneously, while the growth rates of all cells were never increased or reduced in the same sample. The kinetic responses of the DPD2794 and TV1061 were very similar, demonstrating the same toxic pattern (Figure 2A,C). In both strains, the cells’ response patterns were observed only at the highest tested concentration, while each sport supplement affected the bacterial response pattern differently. For example, the luminescence signal in the SS5 and SS7 were inhibited and induced, respectively. Such variations in the cell responses may be influenced by the differences in the sport supplement composition, and indicates that both of these strains are sensitive to such changes. On the other hand, DPD2544 have always demonstrated the same pattern in all of the tested sport supplements, e.g., induction at the lower concentration and inhibition at the higher tested concentrations (Figure 2B). The possible reasons for these uniform responses may be the presence of specific damaging agent/s in all of the compositions that could have induced such an effect in this strain.

## 4. Materials and Methods

### 4.1. Materials

LB Broth (L3022) Lennox L Broth (10 g/L Tryptone; 5 g/L Yeast Extract; 5 g/L NaCl); LB Broth with agar (L2897) Lennox (Powder microbial growth medium); Kanamycin (K1876) disulfate salt from *Streptomyces kanamyceticus* (amino-glycoside antibiotic), Sucralose ≥98% (HPLC) (69293), Saccharin ≥99% (240931), Advantame (80054), Neotame (49777) and Acesulfame Potassium-K (European Pharmacopoeia (EP) Reference Standard) (A0070000) were purchased from Sigma-Aldrich (Sigma-Aldrich, St. Louis, MO, USA). Aspartame (47135) was purchased from SUPELCO (St. Louis, MO, USA). Ethanol (absolute for analysis EMSURE^®^ ACS, ISO, Reag, Ph Eur) was purchased from Merck Millipore (Burlington, MA, USA) (1.00983.2500). A variety of ten sport supplements containing artificial sweeteners were purchased from a local vendor. The concentrations range chosen for the pure artificial sweeteners samples, was based on the FDA acceptable daily intake (ADI). ADI is calculated as milligrams per kilogram body weight per day (mg/kg bw/d): ace-k (15), advantame (32.8), aspartame (50), neotame (0.3), saccharin (15) and sucralose (5). In addition, for the sport supplements samples, the recommended amount for consumption (as instructed by the company), is detailed in Table 3, and was considered for the choice of the samples’ concentrations range.

### 4.2. Bioluminescent Bacteria from E. coli

The *Escherichia coli* strains used in this study, *E. coli* TV1061, DPD 2544 and DPD2794, were obtained from S. Belkin (Hebrew University, Jerusalem, Israel) (see Table 4). The strains harbor a plasmid-borne fusion of the different Promoters to a reporter gene [85]. The promoter is chromosomally integrated to the reporter operon, which has five promotor-less structural genes responsible for both the heterodimeric luciferase units (lux A and B) and the synthesis of the luciferase substrate, tetradecanal, by an ATP-and NADPH-dependent multi-enzyme complex composed of fatty acid reductase, transferase, and synthetase (lux C, D and E) [53]. The strain stocks were stored at −80 °C with 20% (*v*/*v*) of glycerol, as a cell cryoprotectant additive. The bioreporter strains from the stock solution were placed on LB-agar plates (10 g/L Tryptone; 5 g/L Yeast Extract; 5 g/L NaCl) supplemented with 50 μg/mL kanamycin and, after incubation for two days at 37 °C in an incubator (Binder, Camarillo, CA, USA), they were stored at 4 °C for future experiments.

### 4.3. Growth Conditions

**Figure 3:**
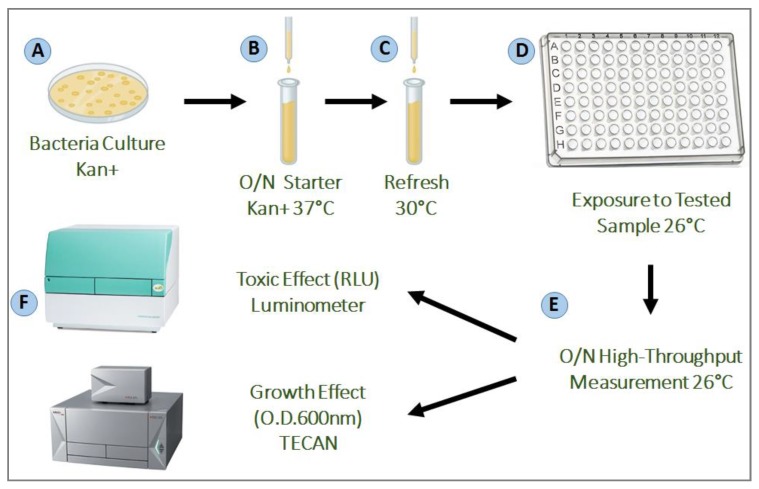
Experimental process. (**A**) each bacteria strain tested was striked on an agar plate containing Kanamycin, and incubated overnight at 37 °C; (**B**) a starter was grown from a single colony from the striked plate, and incubated overnight at 37 °C in a shaking incubator; (**C**) the starter was refreshed by adding 200 μL of the overnight culture into 10 mL of fresh LB, and then grown for 3–4 h at 30 °C in a non-shaking incubator; (**D**) the bacteria strains were then exposed to the different samples of different concentrations in a high-throughput measurement using a 96-well plate; (**E**,**F**) the toxicity (Relative Light Unit (RLU)) and growth (O.D. 600 nm) signals were measured continuously during the 16 h incubation at 26 °C, in the Luminometer and TECAN reader, respectively.

Bacterial cultivation prior to measurements was performed in 10 mL LB medium (10 g/L Tryptone; 5 g/L Yeast Extract; 5 g/L NaCl). Cells were grown overnight at 37 °C in a shaking incubator (NB-205LF, N-BIOTEK, SciMed (Asia) Pte Ltd., Singapore) at 120 rpm. Cultures were then diluted to approximately $10^{7}$ cells/mL and re-grown in 10 mL LB at 30 °C without shaking, until an early exponential phase (Optical Density (O.D.) 600 nm of 0.2), as determined by a UVmini-1240, UV-VIS spectrophotometer (Shimadzu, Singapore) (Figure 3).

### 4.4. Bioluminescence Assay

Bioluminescence activity was measured using a Luminoskan Ascent Luminometer (Thermo Fisher Scientific, Waltham, MA, USA). Measurements took place in white 96-well microtiter plates (NUNC) containing 90 µL of the bacterial culture at OD_600_ = 0.2. Different concentrations of the tested artificial sweeteners or sport supplements were added in volumes of 10 µL to each well (*n* = 3 for each concentration). The negative control was obtained by adding 10 µL LB to the bacteria culture. Moreover, positive control was obtained by the addition of: 2% (*v*/*v*) ethanol, 0.52 mM phenol and 800 ppb Mythomycin C as the known inducers for the following bacterial strains: TV1061, DPD2544 and DPD2794, respectively [53,88]. The artificial sweeteners or sport supplements concentrations range used for the toxicity evaluation is dependent on the specific solubility properties of each tested agent and is described in Table 1. During measurements (16 h), sample temperature was maintained at 26 °C and the plates were continuously shaken. Luminescence values are presented in relative light units (RLU) (Figure 3).

### 4.5. Growth Assay

The effect of the artificial sweeteners and sport supplements on the bacterial growth rates was tested using the TECAN Infinite M200 PRO, City, Switzerland. Measurements took place in transparent 96-well microtiter plates (NUNC) containing 90 µL of the bacterial culture at OD_600_ = 0.2. Different concentrations of the tested artificial sweeteners or sport supplements were added in volumes of 10 µL to each well (*n* = 3 for each concentration). The negative control was obtained by adding 10 µL LB to the bacteria culture. Moreover, positive control was obtained by the addition of: 2% (*v*/*v*) ethanol, 0.52 mM phenol and 800 ppb Mythomycin C as the known inducer for the following bacterial strains: TV1061, DPD2544 and DPD2794, respectively [53,88]. During measurements (16 h), sample temperature was maintained at 26 °C and the plates were continuously shaken. Bacterial growth values are presented in growth relative area AUC (under the curve) (GRA) (Figure 3).

### 4.6. Data Analysis

The bioluminescence signal relating the bacterial response to the different artificial sweeteners and sport supplements was expressed as toxicity index (TI), which was calculated using the formula TI = ((B_S_/B_C_)−1), where B_S_ is the average bioluminescent signal from the tested sample, either artificial sweeteners or sport supplements, and B_C_ is the average bioluminescent signal from the control. Based on the results, a range of values was defined for better analysis of the toxicity effect: if TI ≥ 0.1, a toxic inducing pattern is recognized, if TI ≤ −0.4, a toxic inhibiting pattern is recognized, and, if −0.4 < TI < 0.1, then no toxic effect is found. An additional two toxicity related parameters were determined as follows: MLIC—Minimum Luminescent Inhibition Concentration; MLIndC—Minimum Luminescent Induction Concentration. Growth Relative AUC (area under the curve) (GRA) was calculated using the following formula GRA = (GRA_S_/GRA_C_) × 100, where GRA_S_ is the area under the growth curve from the tested sample, either artificial sweetener or sport supplements and GRA_C_ is the area under the growth curve from the control. Based on the results, a range of values was defined for better analysis of the growth effect: if GRA ≥ 120%, a growth inducing pattern is recognized, if GRA ≤ 80%, a growth inhibiting pattern is recognized and, if 80% < GRA < 120%, then no growth effect is found. An additional two growth related parameters were determined as follows: MGIC—Minimum Growth Inhibition Concentration; MGIndC—Minimum Growth Induction Concentration.

## 5. Conclusions

The toxicity effect of six artificial sweeteners and ten sports supplements was evaluated by the exposure to a bioreporter panel, which consists of three different bioluminescent bacterial strains (*E. coli*), i.e., cytotoxic (TV1061), genotoxic (DP2794) and strain sensitive to membrane damage agents (DPD2544). The differences in the cells’ response patterns did not only provide information about the possible toxicity effect of these additives, but also allowed the creation of a specific response pattern which may be used in future studies. Furthermore, the type of toxicity determined by the proposed system was similar to the information found in literature, suggesting the efficiency of the proposed system for fast and sensitive toxicity evaluation. Similarly, with the artificial sweeteners, the bioreporter panel responded with different response patterns to the ten sports supplements tested in this study. While some similarities were found in the cells’ responses to the artificial sweeteners, the complicated sport supplements composition limit our understanding and information about the actual role of the artificial sweetener addition. However, the triggered luminescent and affected growth rates indicate that all tested sport supplements were toxic to the bacteria. The induction and inhibition effects on the DPD2544 strain suggest that the primary mode of action of these mixtures was damaging the cellular membrane. Moreover, *E. coli* is an indigenous gastro intestinal microorganism, and serves as a model for the gut bacteria. The human colonic microbiome is a complex microbial community that has a significant impact on individual’s health. This is a diverse community that reaches high cell densities and includes dominant phyla including Bacteroidetes, Firmicutes, Actinobacteria and Proteobacteria [89,90]. The indigenous gastrointestinal tract microflora has profound effects on the anatomical, physiological and immunological development of the host [91]. In this study, we demonstrated the toxicity effect on *E. coli* in vitro. With this consideration, we may speculate that the response observed in our study may be relevant to gut microbiome and thus may influence human health. Moreover, since artificial sweeteners are resistant to wastewater treatment processes [33], they have been identified as emerging environmental pollutants [31,32]. Several environmental studies have confirmed their distribution in the water cycle [34,35,36,37,38,39], with ace-k and sucralose concentrations of up to the μg L^−1^ range [31,40]. In this study, sucralose repressed luminescence in all the bioreporter bacteria, the highest inhibition effect was observed with TV1061 strain (1 mg/mL), then with DPD2544 (50 mg/mL) and DPD2794 (100 mg/mL). In addition, ace-K induced luminescence only in genotoxic sensitive bacteria (DPD2794) indicating its possible genotoxicity. The tested bioluminescent bacterial panel can potentially be used for detecting artificial sweeteners in the environment.

## Figures and Tables

**Table 1:** Artificial sweeteners toxicity and viability effect (mg/mL).

	Strain	MLIC	MLIndC	MGIC	MGIndC
**Aspartame**	TV1061	N.E.	N.E.	N.E.	N.E.
	DPD2544	N.E.	N.E.	N.E.	N.E.
	DPD2794	N.E.	4	N.E.	N.E.
**Sucralose**	TV1061	1	N.E.	50	N.E.
	DPD2544	50	N.E.	50	N.E.
	DPD2794	100	N.E.	50	N.E.
**Saccharin**	TV1061	N.E.	5	5	N.E.
	DPD2544	N.E.	N.E.	N.E.	N.E.
	DPD2794	N.E.	5	N.E.	N.E.
**Advantame**	TV1061	N.E.	N.E.	N.E.	2
	DPD2544	N.E.	N.E.	N.E.	N.E.
	DPD2794	N.E.	N.E.	N.E.	N.E.
**Neotame**	TV1061	N.E.	2	N.E.	N.E.
	DPD2544	2	N.E.	N.E.	N.E.
	DPD2794	N.E.	N.E.	N.E.	N.E.
**Ace-K**	TV1061	N.E.	N.E.	N.E.	N.E.
	DPD2544	N.E.	N.E.	N.E.	N.E.
	DPD2794	N.E.	10	N.E.	N.E.

MLIC—Minimum Luminescent Inhibition Concentration; MLIndC—Minimum Luminescent Induction Concentration; MGIC—Minimum Growth Inhibition Concentration; MGIndC—Minimum Growth Induction Concentration; N.E.—No Effect.

**Table 2:** Sport supplements’ toxicity and viability effect (µg/mL).

	Strain	MLIC	MLIndC	MGIC	MGIndC
**SS1**	TV1061	N.E.	2000	N.E.	N.E.
	DPD2544	2 × 10^−3^	2 × 10^−6^	N.E.	N.E.
	DPD2794	N.E.	N.E.	2000	N.E.
**SS2**	TV1061	N.E.	N.E.	N.E.	N.E.
	DPD2544	1 × 10^−3^	1 × 10^−6^	N.E.	N.E.
	DPD2794	N.E.	N.E.	1000	N.E.
**SS3**	TV1061	N.E.	N.E.	N.E.	4000
	DPD2544	4 × 10^−3^	4 × 10^−6^	N.E.	N.E.
	DPD2794	4000	N.E.	N.E.	4000
**SS4**	TV1061	N.E.	N.E.	N.E.	5000
	DPD2544	5 × 10^−3^	5 × 10^−6^	N.E.	N.E.
	DPD2794	N.E.	N.E.	N.E.	5000
**SS5**	TV1061	N.E.	5000	N.E.	N.E.
	DPD2544	5 × 10^−3^	5 × 10^−6^	N.E.	N.E.
	DPD2794	N.E.	5000	5000	N.E.
**SS6**	TV1061	N.E.	N.E.	N.E.	3000
	DPD2544	3 × 10^−3^	3 × 10^−6^	N.E.	N.E.
	DPD2794	N.E.	N.E.	N.E.	N.E.
**SS7**	TV1061	5000	500	5000	N.E.
	DPD2544	5 × 10^−3^	5 × 10^−6^	N.E.	N.E.
	DPD2794	5000	N.E.	5000	N.E.
**SS8**	TV1061	N.E.	N.E.	N.E.	2000
	DPD2544	2 × 10^−3^	2 × 10^−6^	N.E.	N.E.
	DPD2794	N.E.	N.E.	N.E.	2000
**SS9**	TV1061	N.E.	3000	N.E.	N.E.
	DPD2544	3 × 10^−3^	3 × 10^−6^	N.E.	N.E.
	DPD2794	N.E.	N.E.	N.E.	N.E.
**SS10**	TV1061	N.E.	N.E.	N.E.	3000
	DPD2544	3 × 10^−3^	3 × 10^−6^	N.E.	N.E.
	DPD2794	N.E.	N.E.	N.E.	3000

MLIC—Minimum Luminescent Inhibition Concentration; MLIndC—Minimum Luminescent Induction Concentration; MGIC—Minimum Growth Inhibition Concentration; MGIndC—Minimum Growth Induction Concentration; N.E.—No Effect.

**Table 3:** Sport supplement profile.

	Artificial Sweeteners Content	Recommended Amount for Consumption (1 oz = 30 mL)	Ingredients
**SS1**	Sucralose	2 tablets (5 g), recommended to drink a lot of water	Creatine Hydrochloride, Cellulose, Dicalcium phosphate, Enteric Coating (Cellulose, Sodium Alginate, Medium Chain Triglycerides, Oleic and Stearic Acid), Natural Mint Flavor, Sucralose, Titanium Dioxide
**SS2**	Acesulfame Potassium-K and Sucralose	2 (7 g) to 8 (28 g) scoops in 8–10 oz per serving (2 scoops)	Black Tea Extract, Green Tea Extract, Green Coffee Extract, Micronized Taurine, Micronized l-Glutamine, Micronized l-Arginine, Micronized l-Leucine, Beta-Alanine (as CarnoSyn^®^), Micronized Citrulline, Micronized l-Isoleucine, Micronized l-Valine, Micronized l-Tyrosine, Micronized l-Histidine, Micronized l-Lysine, Micronized l-Phenylalanine, Micronized l-Threonine, Micronized l-Methionine Other Ingredients: Inulin, Acesulfame Potassium, Citric Acid, FD&C Red #40, Malic Acid, Natural and Artificial Flavors, Sucralose, Silion Dioxide
**SS3**	Acesulfame Potassium-K and Sucralose	1 (31 g) to 2 (62 g) scoops in 6–8 oz per scoop	Calcium, Cholesterol, Dietary Fibers, Potassium, Protein, Saturated Fat, Sodium, Sugars, Trans Fat Other Ingredients: Acesulfame Potassium, Cocoa (Processed with Alkali), Enzyme Blend (Aminogen^®^, Lactase), Lecithin, Natural and Artificial Flavors, Salt, Sucralose, Whey Protein Blend (Whey Protein Isolate, Whey Protein Concentrate, Whey Protein Hydrolysate), Xanthan Gum
**SS4**	Sucralose	1 (31 g) to 2 (62 g) scoops in 4–10 oz per scoop	Calcium, Cholesterol, proteins, Sodium, Saturated Fat, sugars, Trans Fat Other Ingredients: Citric Acid, FD&C Red #40 Lake, Lactase, Sucralose, Natural and Artificial Flavors, Soy Lecithin, Whey Protein Isolate, Whey Protein Concentrate, Whey Peptides
**SS5**	Sucralose	2 (9 g) to 6 (27 g) scoops in 10–12 oz per serving (2 scoops)	Caffeine, Green Tea Extract, Green Coffee Extract, Micronized Taurine, Micronized l-Glutamine, Micronized l-Arginine, Micronized l-Leucine, Beta-Alanine (as CarnoSyn^®^), Micronized Citrulline, Micronized l-Isoleucine, Micronized l-Valine, Micronized l-Tyrosine, Micronized l-Histidine, Micronized l-Lysine HCI, Micronized l-Phenylalanine, Micronized l-Threonine, Micronized l-Methionine Other Ingredients: Calcium Citrate, Calcium Silicate, Citric Acid, Gum Blend (Cellulose Gum, Xanthan Gum, Carrageenan), FD&C Blue #2, FD&C Red #40, Inulin, Lecithin, Malic Acid, Natural and Artificial Flavors, Silicon Dioxide, Sucralose, Tartaric Acid
**SS6**	Acesulfame Potassium-K	1 (29.4 g) rounded scoop in 4–10 oz	Calcium, Protein, Saturated Fat, Sodium, Sugars, Trans Fat Other Ingredients: Acesulfame Potassium, Aminogen^®^, Lactase, Lecithin, Natural and Artificial Flavor, Whey Protein Isolate, Whey Protein Concentrate, Whey Peptides
**SS7**	Acesulfame Potassium-K and Sucralose	1 (49 g) to 2 (98 g) scoops in 6 oz per scoop	Alpha lipoic acid, Calcium, Citric Acid, Creatine Monohydrate, Creatine HCI, Dicalcium Phosphate, Dextrose, l-alanine, l-Isoleucine, l-Leucine, l-Valine, Magnesium Oxide, Potassium, Sodium, Sugar, Taurine, Vitamin B6, Vitamin C, Vitamin B12 Other Ingredients: Acesulfame-Potassium, Dextrose, Ethyl-Cellulose, Glucose Polymers, Modcarb™ [Oat Bran, Amaranth, Quinoa, Buckwheat, Millet, Chia], Natural Flavors, Calcium Silicate, Salt, Sucralose, FD&C Yellow No. 6, Soy Lecithin, FD&C Yellow No. 5, Waxy Maize (Corn Starch), (Cluster Dextrin)
**SS8**	Acesulfame Potassium-K and Sucralose	1 (34 g) scoop in 6 oz water or skim milk	Calcium, Cholesterol, Dietary Fiber, Iron, Protein, Saturated Fat, Sodium, Sugar Other Ingredients: Acesulfame-Potassium, Alkalized Cocoa Powder, Calcium Carbonate, Gum Blend (Cellulose Gum, Xanthan Gum, Carrageenan), Natural and Artificial Flavors, Salt, Soy Lecithin, Sucralose, Sunflower-based Creamer (Sunflower oil, Corn syrup solids, Sodium Caseinate, Mono-Diglycerides, Dipotassium Phosphate, Tocopherols), Tricalcium Phosphate, Whey Protein Isolate, Whey Peptides, whey Protein Concentrate
**SS9**	Acesulfame Potassium-K and Sucralose	1 (32.4 g) to 2 (64.8 g) scoops in 8–12 oz	Calcium, Cholesterol, Dietary Fiber, Iron, Potassium, Protein, Saturated Fat, Sodium, Sugar, Trans Fat, Vitamin A, Vitamin C Other Ingredients: Acesulfame-Potassium, Amino Matrix (l-Glycine, l-Taurine, BCAAs (Leucine, Iso-Leucine, Valine), l-Glutamine), Flax Seed Oil, Glucose Polymers, Lactase, Natural and Artificial Flavors, Sucralose, Sea Salt, Suspension Matrix (Xanthan Gum, Cellulose Gum, Guar Gum), Whey Protein Concentrate, Whey Protein Isolate, Whey Protein Hydrolysate
**SS10**	Acesulfame Potassium-K and Sucralose	1 (34.9 g) to 2 (69.8 g) scoops in 8–12 oz	Calcium, Cholesterol, Dietary Fiber, Iron, Multi-level Amino Acid Growth Matrix, Potassium, Protein, Saturated Fat, Sodium, Trans Fat Other Ingredients: Alanine, Arginine, Aspartic Acid, BCAAs (l-Leucine, l-isoleucine, l-Glutamine, l-valine), Cystine, Digestive Enzyme Blend, Egg Albumen, Glycine, Histidine, Lactase, Lysine, Methionine, Micellar Casein, Partially-hydrolyzed Whey Concentrate, Phenylalanine, Proline, Protease, Serine, Tyrosine, Threonine, Tryptophan, Whey Protein Isolate, Whey Protein Concentrate

**Table 4:** Bioluminescent bacterial strains.

Strain	*E. coli* Host Strain	Promoter	Plasmid	Stress Sensitivity	Reference
**TV1061**	RFM443	*grp E*	pGrpELux5	Heat Shock (Cytotoxic)	[86]
**DPD2544**	W3110	*fab A*	pFabALux6	Fatty Acid Availability (Cytotoxic)	[53]
**DPD2794**	RFM443	*rec A*	pRecALux3	SOS—DNA Damage (Genotoxicity)	[87]

## Supplementary Materials

The Supplementary Materials are available online.
