# Supplementary material for: Measuring Artificial Sweeteners Toxicity Using a Bioluminescent Bacterial Panel
Source: Molecules. 2018 Sep 25;23(10):2454. doi: 10.3390/molecules23102454 (PMC6222326; doi:10.3390/molecules23102454)
Supplement: Supplementary file 1 [file molecules-23-02454-s001.zip › molecules-346630-SI.docx]

*Supplementary Data*

**Measuring Artificial Sweeteners Toxicity using a Bioluminescent Bacterial Panel**

| 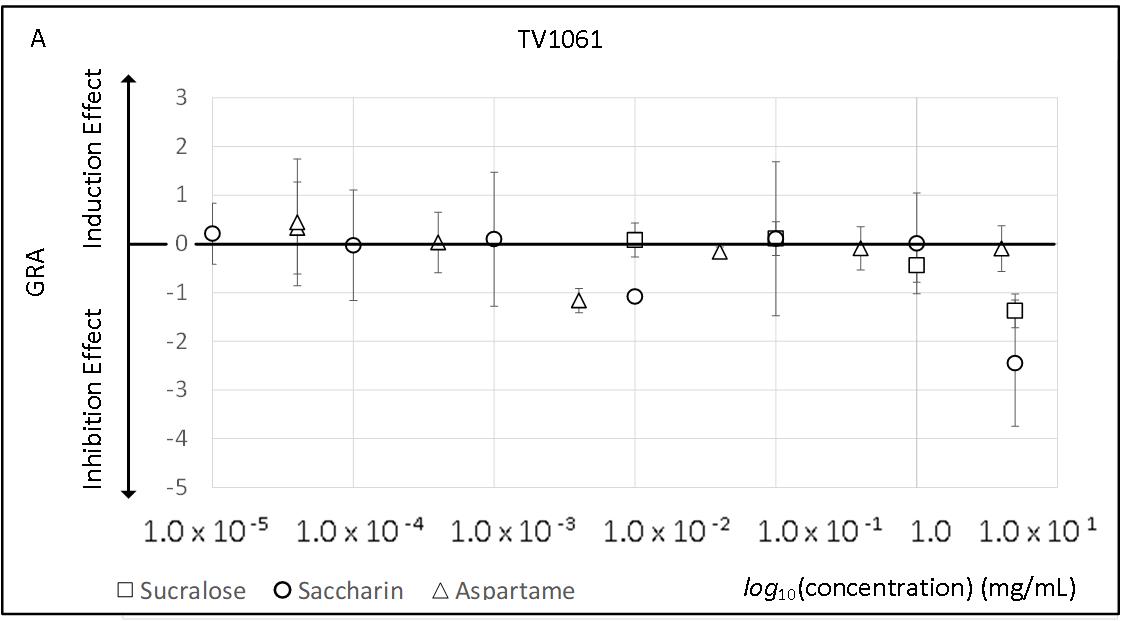 |
| --- |
| 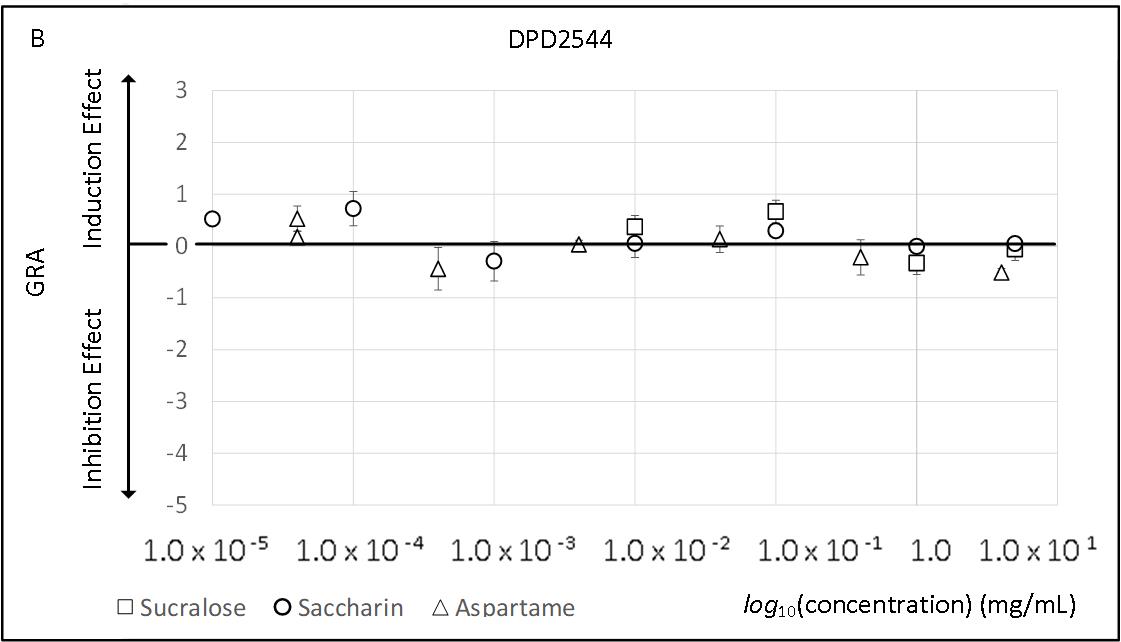 |
| 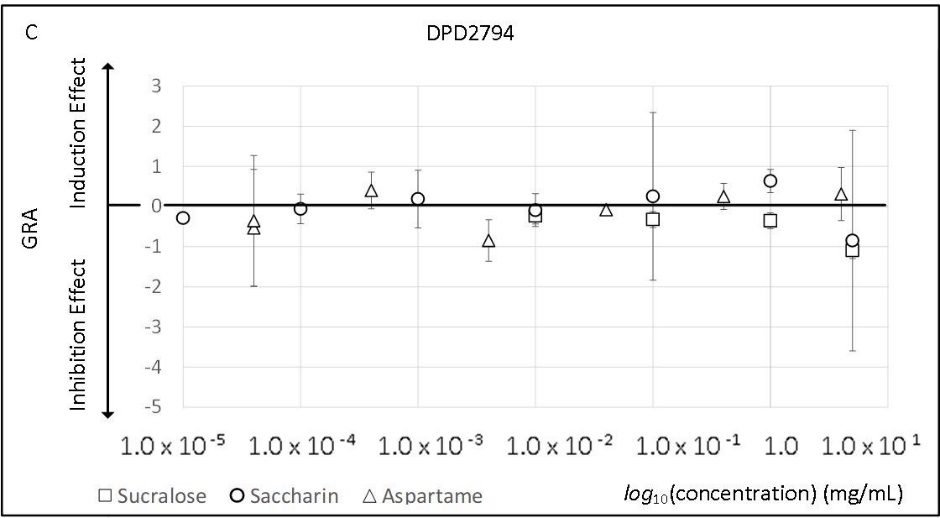 |

**Figure 1S.** Artificial Sweeteners Growth Influence. Growth Relative Area-Under-Curve (AUC) (GRA) of different artificial sweeteners: Sucralose, Saccharin and Aspartame on the three tested bioluminescent bacteria strains: A) TV1061, B) DPD2544 and C) DPD2794.

| 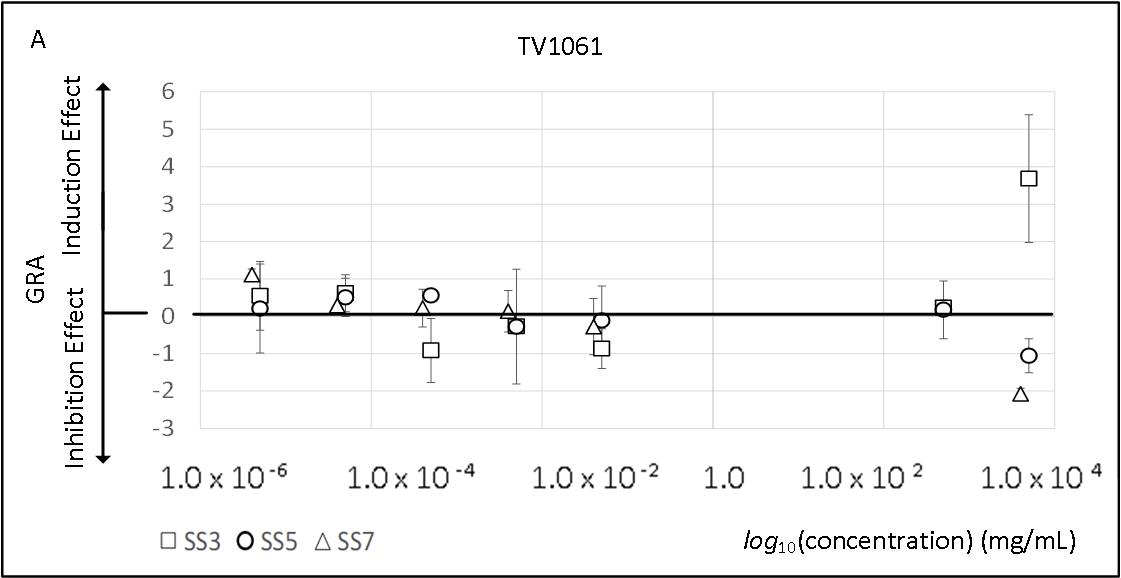 |
| --- |
| 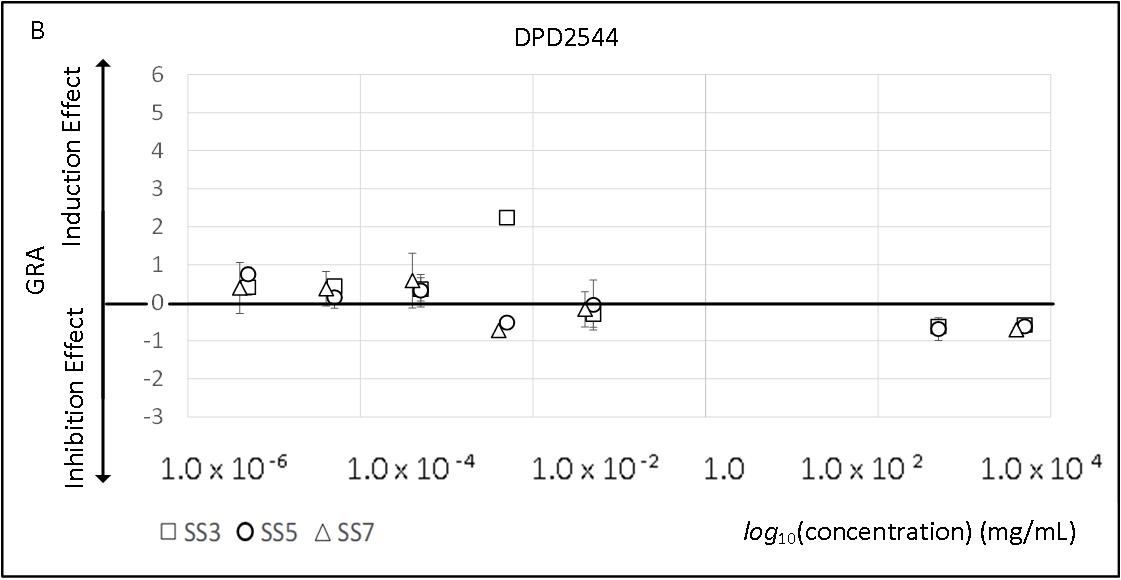 |
| 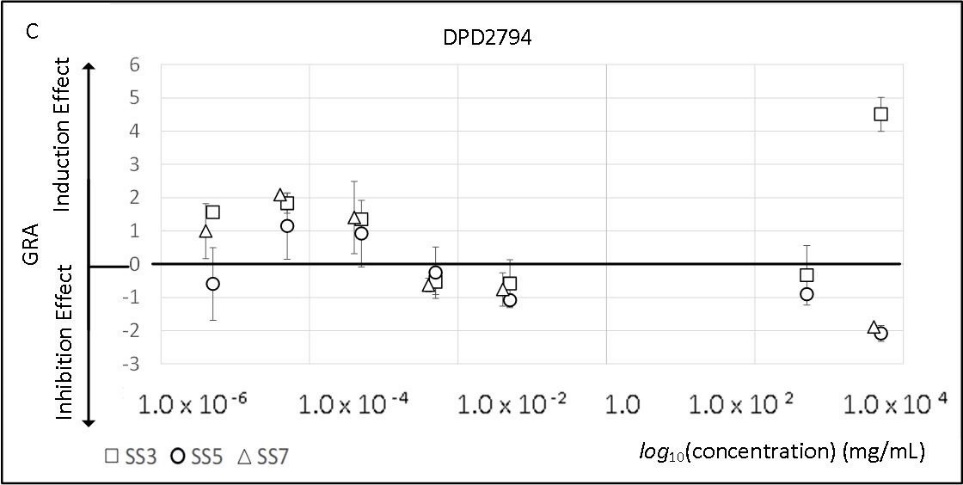 |

**Figure 2S.** Sport Supplements Growth Influence. Growth Relative Area-Under-Curve (AUC) (GRA) of different artificial sweeteners: SS3, SS5 and SS7 on the three tested bioluminescent bacteria strains: A) TV1061, B) DPD2544 and C) DPD2794.
